# Pilot Study of PIVKA-II in the Prognostic Assessment of Hepatocellular Carcinoma in Chronic Viral Hepatitis: Comparative Findings from HBV and HCV Cohorts from a Single Center in Serbia

**DOI:** 10.3390/biomedicines13112653

**Published:** 2025-10-29

**Authors:** Ivana Milošević, Nataša Nikolić, Sanja Stanković, Ana Filipović, Jovana Ranin, Irena Paunović, Jelena Simić, Branko Beronja

**Affiliations:** 1 Clinic for Infectious and Tropical Diseases, University Clinical Center of Serbia, Bulevar Oslobođenja 16, 11000 Belgrade, Serbia; ivana.milosevic@med.bg.ac.rs (I.M.); nanika1984@yahoo.com (N.N.); anafilipovic0211@gmail.com (A.F.); jovana.ranin@hotmail.rs (J.R.); irenapaunovic12@gmail.com (I.P.); simicj093@gmail.com (J.S.); 2 Faculty of Medicine, University of Belgrade, Dr Subotica 8, 11000 Belgrade, Serbia; 3 Center for Medical Biochemistry, University Clinical Center of Serbia, 11000 Belgrade, Serbia; sanjast2013@gmail.com; 4 Faculty of Medical Sciences, University of Kragujevac, 34000 Kragujevac, Serbia

**Keywords:** protein induced by vitamin K absence or antagonist-II, alpha-fetoprotein, viral hepatitis, hepatocellular carcinoma

## Abstract

**Background:** Hepatocellular carcinoma (HCC) frequently develops in patients with chronic hepatitis B and C. Early detection is critical, but current methods, including ultrasound and AFP, have suboptimal accuracy. Objectives: This study aimed to evaluate the predictive performance of protein induced by vitamin K absence or antagonist-II (PIVKA-II) and alpha-fetoprotein (AFP) testing, alone and in combination, for HCC development. **Methods:** A retrospective cohort study at a single university center included 242 CHB and 181 CHC patients. Data on demographics, clinical status, laboratory parameters, and imaging were collected, with fibrosis and steatosis assessed by FibroScan^®^. Serum AFP and PIVKA-II were measured, but measurements of PIVKA-II in patients receiving vitamin K antagonists were excluded from the analysis. HCC diagnosis and staging followed clinical guidelines. Cox regression and ROC analyses identified independent predictors and evaluated biomarker accuracy for HCC detection. **Results:** HCC incidence was comparable between cohorts (5.0% in CHB vs. 5.5% in CHC). Both AFP and PIVKA-II independently predicted HCC development in multivariate models adjusted for age and sex. The combined biomarker score (AFP × PIVKA-II) showed superior predictive accuracy with hazard ratios of 1.38 (CHB) and 1.36 (CHC). ROC analyses demonstrated high discriminative ability for PIVKA-II (AUC ~0.81) and AFP (AUC ~0.83) in both cohorts. Additional independent predictors were chronic alcohol abuse, cirrhosis, and higher liver stiffness measurements. Specific viral factors such as HBeAg positivity and HCV subgenotype 1b were also associated with increased HCC risk. **Conclusions:** AFP and PIVKA-II are independent, valuable biomarkers for HCC risk in chronic hepatitis B and C. Combined use improves early detection, aiding timely treatment. These results support adding PIVKA-II to AFP in surveillance, but larger studies are needed to confirm the findings and refine cut-off values.

## 1. Introduction

Hepatocellular carcinoma (HCC) is the sixth most commonly diagnosed cancer and the third leading cause of cancer-related death worldwide, accounting for over 900,000 new cases and more than 800,000 deaths annually [1,2]. Historically, chronic viral hepatitis B (CHB) and hepatitis C (CHC) have been among the predominant etiologic factors, together accounting for more than 75% of HCC cases globally. Despite the availability of effective antiviral therapies and the widespread implementation of HBV vaccination programs, chronic viral hepatitis continues to be a major contributor to the global HCC burden, particularly in regions with limited access to screening and sustained virological suppression or response [3]. Furthermore, monitoring for additional risk factors for HCC remains insufficient. This is particularly important given that the burden of viral hepatitis is further compounded by the rising global prevalence of steatotic liver disease and alcohol-associated liver disease, both of which may act synergistically with chronic viral hepatitis to accelerate liver fibrosis progression and increase the risk of hepatocarcinogenesis [4,5,6].

HCC is characterized by poor survival outcomes, largely due to asymptomatic progression and diagnosis at advanced stages when curative treatment options are limited [7]. Early detection is essential for improving prognosis, but current surveillance strategies—based on ultrasound and alpha-fetoprotein (AFP) testing—have suboptimal sensitivity and specificity, especially for early stage tumors [8,9].

Protein induced by vitamin K absence or antagonist-II (PIVKA-II), also known as des-gamma-carboxy prothrombin (DCP), has emerged as a promising biomarker in this setting. PIVKA-II is an abnormal prothrombin molecule produced by malignant hepatocytes due to impaired vitamin K-dependent carboxylation. Its biological role in hepatocarcinogenesis is linked to tumor growth, angiogenesis, and invasion, making it not only a diagnostic but also a relevant marker of HCC progression [10,11,12,13]. Several studies have demonstrated that PIVKA-II, when used in combination with AFP, enhances the accuracy of HCC detection and improves risk stratification compared with either marker alone [14,15,16]. In addition, serum PIVKA-II level was demonstrated to be the most useful parameter predisposing to portal venous invasion, an important factor adversely affecting prognosis in HCC [17]. Importantly, most data come from Asian cohorts, while evidence in European patients with chronic viral hepatitis remains scarce [18].

This study aimed to compare the clinical characteristics of patients with CHB and CHC, with a particular focus on evaluating the predictive value of PIVKA-II and AFP for HCC development. The overarching goal was to assess whether the addition of PIVKA-II could improve risk stratification and support more effective surveillance strategies in patients with chronic viral hepatitis.

## 2. Materials and Methods

This study was a retrospective cohort analysis conducted at a single university clinical center involving 242 patients with CHB and 181 patients with CHC. The study included adults (≥18 years) with confirmed CHB or CHC based on serological and molecular testing. Only patients who had available demographic, clinical, and laboratory data, and who provided informed consent, were included in the analysis. Exclusion criteria were strict to avoid confounding factors; patients with HIV, HBV, HCV, or HDV co-infection were excluded. Additionally, patients with a prior diagnosis of HCC or those who had received prior chemotherapy, immunotherapy, or other cancer-directed treatments were excluded from the study. Importantly, patients on vitamin K antagonists (VKAs) were excluded from the analysis of PIVKA-II levels, as these medications interfere with the measurement of vitamin K-dependent proteins and could therefore influence the results. Patients with incomplete medical records or missing key data related to baseline liver function, fibrosis stages, or biomarker measurements were also excluded.

Demographic information, including age and sex, were obtained from medical records. Behavioral factors such as smoking, alcohol consumption, and drug use (intravenous and non-intravenous) were documented through patient questionnaires. Chronic alcohol abuse was classified according to the World Health Organization (WHO) guidelines, and current smoking was defined as smoking within the past 30 days. Intravenous drug use (IVDU) was particularly noted as a risk factor for both viral hepatitis and liver disease progression.

Liver fibrosis and steatosis were assessed using FibroScan^®^ (Echosens, Paris, France), a non-invasive vibration-controlled transient elastography (VCTE) technique that simultaneously measures liver stiffness (LSM, in kilopascals) and the controlled attenuation parameter (CAP, in dB/m). Interpretation of liver stiffness values was based on the EASL Clinical Practice Guidelines (2021), with etiology-specific cut-offs applied. For chronic hepatitis B (CHB), significant fibrosis (≥F2) was defined as LSM ≥ 7.9 kPa, advanced fibrosis (≥F3) as ≥8.8 kPa, and cirrhosis (F4) as ≥11.7 kPa. For chronic hepatitis C (CHC), the corresponding thresholds were ≥7.2 kPa for ≥F2, ≥9.5 kPa for ≥F3, and ≥12.5 kPa for cirrhosis. Steatosis grading was based on the CAP values, with ≥238 dB/m indicating mild steatosis (S1), ≥260 dB/m moderate steatosis (S2), and ≥290 dB/m severe steatosis (S3), regardless of underlying liver disease etiology [11]. Cirrhotic patients were further categorized according to the Child–Pugh classification, which assesses liver function and complications.

Molecular analyses included the measurement of HBV DNA in CHB patients and HCV RNA in CHC patients using standard real-time PCR assays on the Abbott m2000 RealTime System (Abbott Laboratories^®^, Chicago, IL, USA). Serum levels of AFP and PIVKA-II were quantified using commercially available enzyme-linked immunosorbent assays: AFP was determined using the Abbott assay (Abbott Laboratories^®^, Chicago, IL, USA), and PIVKA-II was measured using the Roche Diagnostics assay (Roche Diagnostics^®^, Basel, Switzerland).The use of these biomarkers was based on the current recommendations from the European Association for the Study of the Liver (EASL) and the Japan Society of Hepatology (JSH), which support their role in the early detection and risk assessment of HCC [19,20,21]. Data addressing the patients’ comorbidities (hypertension, diabetes mellitus, cardiovascular, renal, neurological and psychiatric disorders) were gathered using medical records. Hepatic complications such as ascites, hepatic encephalopathy, and portal hypertension were also recorded. Treatment approaches, including surgical resection, microwave ablation, transarterial chemoembolization (TACE), and sorafenib therapy, were noted for patients diagnosed with HCC.

For CHB patients, antiviral treatment regimens included tenofovir disoproxil fumarate (TDF) (94 patients, 38.8%) and tenofovir alafenamide (TAF) (67 patients, 27.6%). For CHC patients, antiviral treatments included sofosbuvir/velpatasvir (78 patients, 43.1%), glecaprevir/pibrentasvir (92 patients, 50.8%), and elbasvir/grazoprevir (11 patients, 6.1%). Patients with prior exposure to pegylated interferon (PEG-IFN) were also included as long as they met the study’s inclusion criteria. All patients were closely monitored for adherence to treatment as well as for viral suppression and liver function.

The diagnosis of HCC was established in accordance with the EASL Clinical Practice Guidelines on the management of hepatocellular carcinoma [20]. HCC staging followed the Barcelona Clinic Liver Cancer (BCLC) system [22]. All patients underwent serum AFP and PIVKA-II testing as part of routine surveillance. In individuals at risk for HCC, detection of a focal liver lesion ≥ 10 mm on the surveillance ultrasound prompted further assessment with contrast-enhanced multiphasic computed tomography (CT) or magnetic resonance imaging (MRI). The diagnosis of HCC was confirmed non-invasively in cases where lesions demonstrated the characteristic radiological hallmarks of arterial phase hyperenhancement (APHE) followed by washout in the portal venous or delayed phases. If imaging was inconclusive, percutaneous liver biopsy was performed for histopathological confirmation. All imaging and diagnostic procedures were interpreted by experienced hepatologists and radiologists specialized in liver disease.

In patients exhibiting elevated serum levels of AFP and/or PIVKA-II, supplementary imaging, typically contrast-enhanced multiphasic CT or MRI, was undertaken to confirm or exclude HCC in accordance with the guideline-based diagnostic criteria. The biomarkers PIVKA-II and AFP were analyzed to evaluate their predictive role for HCC development. The combined effect of PIVKA-II and AFP was also evaluated by calculating a composite variable derived from the product of the two biomarker values (PIVKA-II × AFP).

Statistical analyses were performed using IBM SPSS Statistics, version 23.0 (IBM Corp, Armonk, NY, USA). Prior to inferential testing, the distribution of all scalar (continuous) variables was assessed for normality using the Kolmogorov–Smirnov and Shapiro–Wilk tests. Variables with a normal distribution were expressed as means with standard deviations (SD), while non-normally distributed variables were presented as medians with interquartile ranges (IQR). Categorical variables were reported as absolute and relative frequencies. For comparisons between the CHB and CHC cohorts, the independent samples t-test was used for normally distributed continuous variables, and the Mann–Whitney U test for non-normally distributed variables. The Chi-square test or Fisher’s exact test, as appropriate, was applied for categorical data.

Univariate Cox proportional hazards regression analysis was performed to identify variables associated with the development of hepatocellular carcinoma (HCC). Variables with *p*-values < 0.05 as well as those approaching statistical significance (*p* < 0.125) were subsequently included in the multivariate Cox regression model to identify independent predictors of HCC occurrence. In instances where no variables reached statistical significance in univariate analysis, all candidate variables were included in the multivariate model. A total of six regression models were constructed for the purposes of this study, each adjusted for patient age and sex. To assess the discriminatory performance of individual biomarkers, including PIVKA-II and AFP, receiver operating characteristic (ROC) curve analysis was conducted. The area under the curve (AUC) was calculated to evaluate predictive accuracy, with values approaching 1.0 indicating greater discriminatory power. To assess the discriminatory performance of AFP and PIVKA-II, ROC curve analysis was performed. Cut-off values were determined by identifying the threshold that maximized the Youden index (sensitivity + specificity − 1), representing the point of optimal diagnostic accuracy in our sample. This approach ensured that the selected thresholds were not arbitrarily chosen but statistically derived based on the characteristics of the study population. All statistical tests were two-sided, and a *p*-value of less than 0.05 was considered statistically significant.

This study was conducted in accordance with the principles of the Declaration of Helsinki. The study protocol was reviewed and approved by the Ethics Committee of the University Clinical Center of Serbia where the patients were treated (Approval No. 1228/20). Given the retrospective design, informed consent was obtained, and all patient data were fully anonymized prior to analysis, ensuring confidentiality and compliance with the institutional and international ethical standards.

## 3. Results

A total of 242 patients with CHB and 181 patients with CHC were compared according to their demographic, behavioral, histological, biochemical, and prognostic parameters. In the CHB cohort, 117 individuals (48.3%) were male and 125 (51.7%) were female, whereas the CHC cohort comprised 106 males (58.6%) and 75 females (41.4%) (*p* = 0.037). No statistically significant difference in age between sexes was observed in either cohort (CHC *p* = 0.510, CHB *p* = 0.684). Mean age did not differ significantly, measuring 53.10 ± 15.33 years in CHB versus 51.31 ± 13.89 years in CHC patients (*p* = 0.254). Body mass index (BMI) was higher among the CHB patients (25.83 ± 4.21 kg/m^2^) compared with the CHC patients (21.92 ± 3.87 kg/m^2^; *p* = 0.043). Chronic alcohol abuse was documented in 21 CHB patients (8.6%) and 34 CHC patients (18.8%; *p* = 0.025), while intravenous drug use occurred in 13 CHB patients (5.3%) versus 67 CHC patients (37.0%; *p* < 0.001). Non-intravenous drug use was noted in 7 CHB (2.9%) and 21 CHC (11.6%) patients (*p* = 0.004). Current smoking prevalence stood at 33.5% in CHB and 45.3% in CHC (*p* = 0.043) (Table 1).

Mild or absent liver fibrosis (F0/F1) was observed in 130 CHB patients (53.9%) and 72 CHC patients (39.8%; *p* = 0.042). Moderate fibrosis to cirrhosis (F2–F4) showed no statistically significant differences between cohorts. CAP values averaged 241.5 ± 29.58 dB/m in CHB and 248.5 ± 32.34 dB/m in CHC (*p* = 0.575). Among the cirrhotic patients, ascites was present in 6 CHB cases (2.5%) versus 11 CHC cases (6.0%; *p* = 0.028), whereas the rates of hepatic encephalopathy, portal hypertension, and esophageal varices did not differ significantly (Table 1).

Hypertension was reported in 72 CHB patients (29.7%) and 64 CHC patients (35.4%; *p* = 0.221), and diabetes mellitus in 34 CHB (14.1%) versus 21 CHC (11.6%; *p* = 0.503). Psychiatric disorders were documented in 13 CHB patients (5.4%) compared with 30 CHC patients (16.6%; *p* = 0.001). All other assessed comorbid conditions (cardiovascular, respiratory, renal, malignant, connective tissue, neurological, thyroid) showed no significant inter-group differences (Table 1). Differences in laboratory parameters between the CHB and CHC cohorts are presented in tabular form (Supplementary Table S1).

Among the CHB patients, the mean HBV DNA level was 15,213.0 ± 5412.0 IU/mL. HBeAg was positive in 61 individuals (25.2%), while anti-HBe antibodies were detected in 180 (74.3%) patients. Regarding therapy, 94 patients (38.8%) received TDF, and 67 (27.6%) received TAF. In the CHC cohort, the mean HCV RNA level was 1,359,520.0 ± 825.2 IU/mL. Genotype distribution followed the pattern commonly reported in European countries, with genotype 1 as the most prevalent and genotype 3 as the second most frequent. Detailed data are presented in Table 2. Antiviral therapy was administered according to the current guidelines, with detailed regimens outlined in Table 2.

### 3.1. Hepatocellular Carcinoma, Stage, Incidence and Survival

HCC developed in 12 (5.0%) CHB patients and 10 CHC patients (5.5%; *p* = 0.697). Mortality among those diagnosed with HCC was 1.2% (3 patients) in CHB and 2.2% (4 patients) in CHC (*p* = 0.970). Mean interval from HCC diagnosis to death was longer in CHB (8.2 ± 4.2 months) than in CHC (7.1 ± 4.1 months; *p* = 0.016). Rates of surgical resection, microwave ablation, TACE, and sorafenib therapy did not differ significantly (Table 3). Distribution across HCC stages I to IV showed no statistically significant differences.

Patients receiving VKAs comprised 12.5% (n = 30) of the CHB cohort and 5.5% (n = 10) of the CHC cohort. This subgroup exhibited significantly elevated PIVKA II levels (385.7 ± 78.7) compared with other patients (118.8 ± 38.9). Notably, no cases of HCC were observed among patients receiving this particular oral anticoagulant therapy. Due to the known interference of VKAs with PIVKA-II measurements, these patients were excluded from the analysis assessing the predictive value of PIVKA-II levels.

### 3.2. Predictive Performance of Biomarkers and Risk Modeling

**Figure 1:**
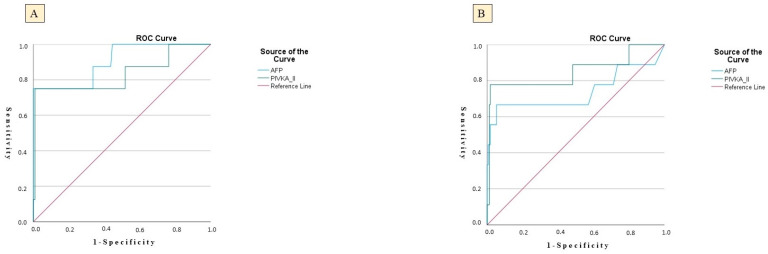
ROC curves showing the diagnostic performance of serum PIVKA-II and AFP for predicting HCC; (**A**) CHC cohort; (**B**) CHB cohort.

In the CHB group, PIVKA-II achieved an AUC of 0.809 (SE = 0.048; *p* = 0.024), indicating substantial ability to differentiate between patients who did and did not develop HCC. At a threshold of 48.5 ng/mL, PIVKA-II demonstrated a sensitivity of 81.5%—correctly identifying 81.5% of eventual HCC cases—and a specificity of 51.5%, reflecting a moderate false-positive rate. The positive predictive value (PPV) for PIVKA-II was 15.18%, while the negative predictive value (NPV) was 98.1%. AFP yielded an even higher AUC of 0.830 (SE = 0.057; *p* = 0.039), suggesting slightly superior discriminative capacity compared with PIVKA-II. Using an AFP cut-off of 11.2 µg/L, sensitivity reached 82.9% and specificity 65.5%, indicating that AFP correctly classified over 80% of HCC outcomes while excluding nearly two-thirds of non-cases. The PPV for AFP was 11.9%, and the NPV was 97.8%, reflecting a more robust ability to exclude non-cases and a moderate ability to predict true positives (Table 4, Figure 1).

Parallel trends were observed in the CHC cohort. PIVKA-II achieved an AUC of 0.812 (SE = 0.046; *p* = 0.012) at a threshold of 47.2 ng/mL, yielding a sensitivity of 82.0% and specificity of 53.9%, mirroring its performance in CHB. The PPV for PIVKA-II in the CHC cohort was 16.23%, while the NPV was 98.1%, indicating a similar predictive performance. AFP in CHC patients produced an AUC of 0.824 (SE = 0.051; *p* = 0.020), with a sensitivity of 83.3% and specificity of 66.5% at a cut-off of 11.6 µg/L. The PPV for AFP was 9.49%, and the NPV was 98.1%, reinforcing AFP’s strong predictive accuracy (Table 4, Figure 1).

### 3.3. HCC Predictors in HBV and HCV Cohorts

In the CHB cohort, the univariate regression analysis (Model 1) revealed that chronic alcohol abuse, severe fibrosis, and cirrhosis were significantly associated with the development of HCC, and these variables were subsequently included in the multivariate model. In the multivariate analysis, chronic alcohol abuse remained an independent predictor of HCC occurrence (HR 2.21; 95% CI 1.74–2.93; *p* = 0.028), corresponding to a 121% increase in HCC risk. In the CHC cohort, univariate analysis identified older age, chronic alcohol abuse, the presence of severe fibrosis, cirrhosis, and higher FibroScan^®^ stiffness values [kPa] as significant predictors of HCC development. In the multivariate model, independent predictors of HCC included older age (HR 1.01; *p* = 0.012), representing a 1% increase in HCC risk per additional year of age; chronic alcohol abuse (HR 1.69; *p* = 0.024), associated with a 69% increase in risk; presence of cirrhosis (HR 3.60; *p* = 0.001), corresponding to a 260% increase in risk; and elevated FibroScan^®^ stiffness values (HR 1.02; *p* = 0.028), with each 1 kPa increase associated with a 2% increase in HCC risk (Table 5).

In Model 2, univariate Cox regression identified age and Child–Pugh class C as significant predictors of HCC development in the CHC cohort. These variables were subsequently included in the multivariate analysis. The multivariate model was adjusted for sex and age. Child–Pugh class C was independently associated with a higher risk of HCC (HR 1.64; 95% CI 1.21–1.81; *p* = 0.041), corresponding to a 64% increased risk compared with patients not in class C. In the CHB cohort, no variable demonstrated statistical significance in the univariate analysis; consequently, all candidate predictors were tested in the multivariate model, where none showed a significant association with HCC development (all *p*-values > 0.05) (Table 5).

In the CHB cohort, the univariate analysis (Model 3) identified the baseline PIVKA-II levels, AFP levels, and combined use of AFP and PIVKA-II levels score as significant predictors of HCC development. In the multivariate model, adjusted for age and sex, both PIVKA-II (HR 1.05; 95% CI 1.00–1.10; *p* = 0.015) and AFP (HR 1.11; 95% CI 1.03–1.20; *p* = 0.006) remained independent predictors of HCC, indicating that each unit increase in PIVKA-II and AFP was associated with a 5% and 11% increase in HCC risk, respectively. Additionally, in the CHB cohort, the combined use of AFP and PIVKA-II showed strong predictive performance, with a hazard ratio of 1.38 (95% CI 1.20–1.46; *p* = 0.001) in the multivariate model, suggesting a 38% increase in HCC risk per unit increase in the combined score (Table 6).

In the CHC cohort, univariate analysis identified the baseline PIVKA-II, AFP, and combined use of AFP and PIVKA-II levels as significant predictors. In the multivariate model adjusted for age and sex, independent predictors of HCC were age, PIVKA-II (HR 1.03; 95% CI 1.01–1.05; *p* = 0.002), and AFP (HR 1.16; 95% CI 1.04–1.24; *p* = 0.012). These results indicate that each unit increase in age, PIVKA-II, AFP, or risk model score was associated with a 4%, 3%, 16%, and 24% increase in HCC risk, respectively. Additionally, in the CHC cohort, the combined use of AFP and PIVKA-II showed strong predictive performance, with a hazard ratio of 1.36 (95% CI 1.21–1.37; *p* = 0.001) in the multivariate model, suggesting a 36% increase in HCC risk per unit increase in the combined score (Table 6).

In the HBV group, HBeAg positivity remained a statistically significant factor (HR 1.56; 95% CI 1.17–2.31; *p* = 0.027), corresponding to a 56% increased risk of HCC. In the HCV cohort, genotype 1b showed a consistent and significant association with HCC risk (HR 1.38; 95% CI 1.02–2.08; *p* = 0.020), corresponding to a 38% increase in risk (Table 6). In univariate models 5 and 6, none of the analyzed variables showed a statistically significant association with the development of HCC in either the CHB or CHC cohorts. All variables included in these models were subsequently analyzed in multivariate Cox regression; however, none remained statistically significant in the adjusted analyses (Supplementary Table S2).

## 4. Discussion

Liver cancer is the third leading cause of cancer-related mortality and the sixth most commonly diagnosed cancer worldwide, with HCC accounting for approximately 90% of all primary liver malignancies [20]. Given that viral hepatitis remains a significant global public health challenge, and that HCC represents one of its most severe long-term complications, this study investigated factors associated with HCC development in patients with CHB and CHC. In addition, it evaluated the sensitivity and specificity of standard surveillance methods—namely ultrasound combined with AFP—and explored the diagnostic performance of PIVKA-II, a biomarker not yet incorporated into routine HCC surveillance protocols in Europe.

Despite advancements in curative and palliative therapies, including surgical resection, liver transplantation, and systemic treatments, HCC prognosis remains poor due to late-stage diagnosis in a large proportion of patients. Early detection is therefore critical and depends on effective surveillance strategies in high-risk populations. Standard surveillance, as recommended by EASL and AASLD, consists of ultrasound with AFP every six months [9,19]. In a meta-analysis, Tzartzeva et al. showed that the addition of AFP to ultrasound significantly improved the sensitivity for detecting early stage HCC, increasing it from 40% with ultrasound alone to 60% with the combined approach [23]. The fact that this strategy misses one in three early stage HCC cases highlights a critical gap in existing surveillance protocols and reinforces the need for enhanced detection tools. The optimal scenario would involve the availability of blood-based biomarkers with adequate diagnostic accuracy to facilitate the early stage detection of HCC. PIVKA-II, also known as des-γ-carboxy prothrombin (DCP), is an abnormal form of prothrombin produced by malignant hepatocytes due to impaired post-translational carboxylation. Unlike normal prothrombin, which requires vitamin K-dependent carboxylation, PIVKA-II lacks γ-carboxyglutamic acid residues, making it a promising tumor marker in HCC [24].

The underlying etiology of HCC should be taken into account, as it can significantly influence the diagnostic performance of both AFP and PIVKA-II [25]. In our study, the diagnostic performance of AFP and PIVKA-II in the CHB cohort was comparable to that observed in the CHC cohort, suggesting that both biomarkers demonstrate similar utility across chronic viral hepatitis etiologies. Our findings demonstrate that both biomarkers possess significant predictive value for HCC development in patients with CHB and CHC, with the AUC values consistently exceeding 0.80 in both cohorts. These findings are consistent with previous reports highlighting the utility of each biomarker in HCC surveillance. Analyzing the available literature on the diagnostic performance of AFP and PIVKA-II in patients with CHB and CHC, PIVKA-II has consistently demonstrated superior accuracy compared with AFP. Liu et al. reported that PIVKA-II alone achieved an AUC of 0.90 for the detection of HCC in HCV-infected patients, outperforming AFP (AUC = 0.80), while the combination of AFP and PIVKA-II further improved the diagnostic accuracy (AUC = 0.93) [26]. Similarly, previous studies have shown that PIVKA-II offers better diagnostic performance than AFP in HBV-related HCC, with reported AUCs of 0.901 for PIVKA-II and 0.765 for AFP [24]. In addition, a large-scale meta-analysis reported pooled AUCs of 0.89 for PIVKA-II and 0.78 for AFP across populations with HBV-related, HCV-related, or mixed-etiology HCC [14].

The findings of this study demonstrate that both AFP and PIVKA-II exhibit high NPV, supporting their clinical utility in ruling out HCC and underscoring their reliability as surveillance tools in at-risk populations. Although higher than the PPV observed for AFP (11.9% in the CHB cohort and 9.49% in the CHC cohort), the PPV for PIVKA-II (15.18% and 16.23% in the CHB and CHC cohorts, respectively) remains suboptimal, highlighting the limitations of relying on a single biomarker for early detection. Parikh et al. emphasized that while AFP and PIVKA-II offer value for the early detection of HCC, neither marker alone provides sufficient sensitivity or specificity to be used as a standalone surveillance tool [8]. PPV of PIVKA-II arises from the low prevalence of HCC in the general population, which is characteristic of screening biomarkers. Although PIVKA-II may exhibit high sensitivity and specificity, its PPV remains limited when applied to cohorts with a low absolute risk such as patients without cirrhosis (81.3% and 72.9% in CHB and CHC cohorts, respectively) or those in the early stages of HCC. The cut-off of 48.5 ng/mL used for PIVKA-II in our CHB cohort yielded a high sensitivity (81.5%), albeit at the expense of reduced specificity, which is consistent with findings from Dong et al., who reported moderate false-positive rates at comparable PIVKA-II thresholds [10]. In contrast, AFP at a cut-off of 11.2 µg/L showed slightly higher specificity (65.5%) with a similar sensitivity (82.9%), suggesting that combining both biomarkers may improve diagnostic accuracy and reduce false positives in HBV related HCC surveillance. This supports the growing body of evidence advocating for dual-marker strategies in high-risk populations to enhance early detection without overburdening follow-up investigations. In comparison to previously published data using a lower AFP cut-off of 8.7 ng/mL, our study used a slightly higher threshold (11.2 µg/L) and achieved a higher sensitivity (82.9% vs. 58%) but lower specificity (65.5% vs. 94%) [27]. While the lower threshold improved the specificity and PPV, it may have resulted in missed early cases. These differences highlight the trade-offs between sensitivity and specificity depending on the chosen cut-off, reflecting the need for context-specific optimization of biomarker thresholds in HCC surveillance strategies for CHB patients.

In the CHC cohort, our findings mirrored the trends observed in CHB. PIVKA-II demonstrated a sensitivity of 82.0% and specificity of 53.9% at a cut-off value of 47.2 ng/mL. AFP performed similarly, with a slightly higher specificity (66.5%) and comparable sensitivity (83.3%) at a cut-off of 11.6 µg/L. Previous evidence supports the role of PIVKA-II as a useful diagnostic biomarker in CHC, consistent with our observations [27]. Although PIVKA-II and AFP demonstrated a high sensitivity and NPV in our HCV cohort, the relatively low specificity and PPV raise concerns about false positives and unnecessary diagnostic follow-up. There is a clear need to establish individualized surveillance strategies in patients with CHC, ensuring that biomarker thresholds are optimized according to clinical context and risk stratification. This limitation reinforces the importance of using a combination of biomarkers to optimize performance. Incorporating dual-marker strategies, such as combining AFP and PIVKA-II, appears to improve sensitivity while balancing specificity.

Our findings indicate that the combination of AFP and PIVKA-II provides superior predictive value for HCC in patients with CHB compared with either biomarker alone. This aligns with the results of Kurniawan et al. and Seo et al. who demonstrated that while AFP and PIVKA-II had similar individual diagnostic performance in differentiating HCC from nonmalignant CHB, their combined use significantly improved the diagnostic accuracy, particularly in cirrhotic patients [28,29]. Notably, both studies identified similar cut-off values for AFP and PIVKA-II, further supporting the consistency and potential clinical applicability of these biomarkers in early HCC detection and risk stratification within CHB cohorts [30].

In our CHC cohort, the combination of AFP and PIVKA-II demonstrated superior predictive performance for HCC compared with the individual use of either marker. These findings are consistent with the study by Liu et al., which highlighted that among the HCV-associated HCC patients, PIVKA-II alone showed the highest individual diagnostic accuracy, and its combination with AFP yielded the best overall performance [26].

In our study, higher liver stiffness values on FibroScan^®^ and the presence of cirrhosis were identified as independent predictors of HCC in the CHC cohort, consistent with the traditionally accepted concept that HCC predominantly arises in the context of end stage liver disease (ESLD) [31,32,33]. Previous studies have shown that HCC in chronic HCV infection develops on a cirrhotic background in 80–90% of cases, while less frequently in non-cirrhotic livers with pronounced inflammation [34,35]. Cirrhosis promotes the development of HCC through chronic hepatocyte injury, regenerative proliferation, fibrotic remodeling, sustained inflammation, oxidative stress, and HCV protein-mediated oncogenic signaling [6,36,37]. Elevated liver stiffness above 12.5–14 kPa reflects advanced fibrosis and indicates increased HCC risk, with values >20–25 kPa correlating with a substantially higher tumor incidence [38,39]. Additionally, chronic alcohol abuse was identified as an independent predictor of HCC in both cohorts. This finding is expected, considering the pathogenic mechanisms through which alcohol promotes tumorigenesis including DNA and protein adduct formation, mutations in tumor suppressor genes (p53, PTEN), activation of oncogenic pathways (c-MYC, β-catenin), oxidative stress, NF-κB and STAT3 signaling, pro-inflammatory cytokine expression (TNF-α, IL-6), and mitochondrial dysfunction [40,41,42]. It is important to emphasize that both alcohol-related liver disease (ALD) and recent antibiotic use may act as confounding factors when interpreting elevated serum PIVKA-II levels [43,44]. Therefore, PIVKA-II measurements in patients with ALD or those treated with antibiotics should be interpreted with caution. In the present study, none of the patients had received antibiotics prior to PIVKA-II testing. However, a proportion of patients had ALD, underscoring the need for further research to establish the optimal PIVKA-II cut-off value for diagnosing hepatocellular HCC in this subgroup. The mechanism by which alcohol affects the serum PIVKA-II levels remains unclear. Although vitamin K deficiency has been proposed as a possible explanation in chronic alcohol users, previous studies have not confirmed a consistent relationship between serum vitamin K concentrations and PIVKA-II levels. This discrepancy underscores a significant gap in understanding and highlights the need for further investigation.

The challenge of establishing a valid cut-off value for PIVKA-II, beyond the factors already mentioned, lies in the fact that other conditions and clinical entities may also lead to elevated levels such as Child–Pugh classes B and C cirrhosis [45]. This subgroup warrants particular attention in HCC screening, as their substantially increased risk of HCC and altered biomarker dynamics may necessitate the definition of distinct cut-off thresholds to ensure optimal diagnostic accuracy. Moreover, elevated PIVKA-II levels have also been reported in association with abnormal bone physiology in females and muscle weakness in males, further underscoring the complexity of interpreting this biomarker [45].

HCV genotypes 1 and 3 predominated in this study, representing the two most common genotypes in Serbia, in line with distribution patterns reported in Europe and globally [46]. This study identified HCV genotype 1b infection as an independent predictor of HCC development, with an HR of 1.38 (95% CI: 1.02–2.08). In an effort to clarify this issue, a prospective study by B. Savino et al., conducted before the availability of DAA therapy, including 163 consecutive HCV-positive patients with cirrhosis, demonstrated that HCV genotype 1b was independently associated with HCC development, with an HR of 3.02 (95% CI: 1.40–6.53) [47]. A meta-analysis by Raimondi S. et al., analyzing 57 studies and focusing on age-adjusted risk estimates, showed that HCV genotype 1b infection is associated with an increased risk of HCC compared with other genotypes, with relative risks ranging from 1.60 to 2.46 depending on the presence of cirrhosis [48]. Considering that our analysis did not evaluate the predictive significance of HCV genotype 1b infection exclusively in patients with cirrhosis, but across all stages of fibrosis, this may explain the somewhat lower HR observed compared with the aforementioned studies.

In Serbia, HBV genotype D is predominant, while a much smaller proportion of patients carry genotype A; other genotypes have not been documented [49]. Importantly, genotype D, similar to genotype C, which is typical for Asia, has been associated with more rapid progression to cirrhosis and HCC, which has important implications for prognosis and clinical management [50]. HBeAg is a crucial marker of active HBV replication and plays a significant role in modulating the host immune response. It can impact both innate and adaptive immune mechanisms, thereby acting as a tolerogen that facilitates HBV persistence within the host [51,52]. Through mechanisms that include immune evasion and interference with apoptotic pathways, HBeAg contributes to the chronicity of HBV infection [51,52]. Furthermore, its ability to evade immune detection and modulate cellular processes may enhance the hepatocarcinogenic potential of HBV, increasing the risk of HCC [53]. HBeAg and its precursors interfere and also stimulate intracellular signaling, which promotes hepatocyte proliferation [53,54]. Together, these mechanisms contribute to key cancer hallmarks, including tumor-promoting inflammation, resistance to cell death, and sustained proliferative signaling, implicating HBeAg as a potential viral oncoprotein [53,54]. The role of HBeAg has been increasingly recognized in relation to disease complications, leading to changes in treatment recommendations. Data support the initiation of NA therapy in all HBeAg-positive patients above the age of 30, as already advised by European guidelines [55]. More recently, Chinese guidelines have even extended these indications to patients younger than 30 years with minimal biochemical activity and no clear clinical evidence of active liver disease [56].

## 5. Conclusions

The results of this study demonstrate that both AFP and PIVKA-II have independent diagnostic value in the detection of HCC among patients with chronic hepatitis B and C. Importantly, the combined use of these two biomarkers yielded superior predictive accuracy compared with either marker alone, consistently across both etiological cohorts.

The study also highlights the need for an individualized approach to patients at risk of HCC, ideally through the development of a scoring system that integrates additional relevant factors, such as HBeAg positivity, HCV genotype and subgenotype, LSM values in kPa, and alcohol abuse, which could be incorporated into existing scoring models. Beyond the additive value of biomarkers, this study highlights that HCC risk is strongly influenced by disease etiology and host-related factors. In the CHC cohort, higher liver stiffness values and the presence of cirrhosis were confirmed as independent predictors of HCC, in line with the long-established association between advanced fibrosis and carcinogenesis. Chronic alcohol consumption emerged as a key risk factor in both the CHB and CHC cohorts, not only contributing to hepatocarcinogenesis through multiple pathogenic mechanisms, but also complicating the interpretation of serum PIVKA-II levels, as alcohol-related liver disease may act as a confounder. In addition, the study identified HCV genotype 1b infection as an independent predictor of HCC development, corroborating previous evidence of its higher oncogenic potential compared with other genotypes. Furthermore, HBeAg positivity was highlighted as a biologically plausible driver of hepatocarcinogenesis due to its immunomodulatory and oncogenic properties, providing additional rationale for guideline-supported initiation of nucleos(t)ide analogue therapy in specific subgroups.

Collectively, these results indicate that future surveillance strategies for HCC should not rely solely on single biomarkers but rather on integrated risk-based models. Such models could incorporate biomarker profiles (AFP, PIVKA-II), viral factors (HBV genotype, HBeAg status, HCV genotype/subgenotype), host-related parameters (liver stiffness, cirrhosis, alcohol exposure), and other clinical cofactors. This approach would allow for the development of individualized scoring systems that are capable of refining surveillance intervals, optimizing cut-off values, and prioritizing high-risk patients for intensified monitoring.

Given the relatively limited sample size, further large-scale, multicenter prospective studies are warranted to validate these findings, standardize biomarker thresholds across diverse populations, and formally integrate AFP and PIVKA-II into international surveillance algorithms. Ultimately, the integration of biomarker-based strategies with clinical and virological risk factors represents a crucial step toward precision surveillance, with the potential to substantially improve early detection and survival outcomes in patients at risk of HCC.

## Figures and Tables

**Table 1:** Overview of the characteristics of the studied cohorts—chronic hepatitis B cohort and chronic hepatitis C cohort.

Variable		CHB Cohort N = 242; *n* (57.2%)	CHC Cohort N = 181; *n* (42.8%)	*p*
Baseline characteristics				
Sex	Male	117 (48.3%)	106 (58.6)	**0.037**
	Female	125 (51.7%)	75 (41.4%)	
Age		53.10 ± 15.33	51.31 ± 13.89	0.254
Vital status (deceased)		4 (1.6%)	3 (1.6%)	0.852
Body mass index [kg/m^2^]		25.83 ± 4.21	21.92 ± 3.87	**0.043**
Chronic alcohol abuse		21 (8.6%)	34 (18.8%)	**0.025**
Intravenous drug use		13 (5.3%)	67 (37.0%)	<**0.001**
Only non-intravenous drug use		7 (2.9%)	21 (11.6%)	**0.004**
Smoking		81 (33.5%)	82 (45.3%)	**0.043**
Liver fibrosis and steatosis parameters				
F0/F1—No/mild fibrosis		130 (53.9%)	72 (39.8%)	**0.042**
F2—Moderate fibrosis		44 (18.3%)	45 (24.9%)	0.186
F3—Severe fibrosis		23 (9.5%)	15 (8.3%%)	0.694
F4—Cirrhosis		45 (18.7%)	49 (27.1%)	0.097
FibroScan^®^ stiffness [kPa]		8.37±7.58	9.17±8.66	0.197
S0—No steatosis		127 (52.5%)	89 (49.1%)	0.510
S1—Mild steatosis		62 (25.6%)	56 (30.9%)	0.287
S2—Moderate steatosis		26 (10.7%)	19 (10.5%)	0.698
S3—Severe steatosis		27 (11.1%)	17 (9.3%)	0.458
FibroScan^®^ CAP [dB/m]		241.5 ± 29.58	248.5 ± 32.34	0.575
Cirrhosis patient profile				
Child–Pugh class A		29 (11.9%)	28 (15.4%)	0.125
Child–Pugh class B		11 (4.5%)	13 (7.1%)	0.059
Child–Pugh class C		5 (2.1%)	8 (4.4%)	0.074
Ascites		6 (2.5%)	11 (6.0%)	**0.028**
Hepatic encephalopathy		4 (1.6%)	4 (2.2%)	0.512
Portal hypertension		15 (6.1%)	17 (9.3%)	0.408
Esophageal varices		15 (6.1%)	16 (8.8%)	0.263
Comorbidities				
Hypertension		72 (29.7%)	64 (35.4%)	0.221
Cerebrovascular insult		5 (2.1%)	3 (1.7%)	0.801
Other cardiovascular diseases		14 (5.8%)	8 (4.4%)	0.572
Diabetes mellitus		34 (14.1%)	21 (11.6%)	0.503
OAK vitamin K antagonist therapy		18 (7.4%)	12 (6.6%)	0.138
Respiratory diseases		14 (5.8%)	13 (7.2%)	0.573
Chronic kidney disease		15 (6.2%)	12 (6.6%)	0.863
Malignant diseases		27 (11.2%)	22 (12.2%)	0.746
Systemic connective tissue diseases		6 (2.5%)	4 (2.2%)	0.891
Neurological diseases		6 (2.5%)	10 (5.5%)	0.083
Hypo/hyperthyroidism		7 (2.9%)	9 (5.0%)	0.261
Psychiatric disorders		13 (5.4%)	30 (16.6%)	**0.001**

Legend: Statistically significant *p*-values (*p* < 0.05) are in bold.

**Table 2:** Virological and therapeutic characteristics of the cohorts.

Variable	CHB Cohort N = 242; *n* (57.2%)	CHC Cohort N = 181; *n* (42.8%)
HBV DNA PCR [IU/mL]	15,213.0 ± 5412.0	n/a
HBeAg positivity	61 (25.2%)	
Anti-HBe positivity	180 (74.3%)	
TDF therapy	94 (38.8%)	
TAF therapy	67 (27.6%)	
HCV RNA RT PCR [IU/mL]	n/a	1,359,520.0 ± 825.2
Genotype 1a		67 (37.0%)
Genotype 1b		14 (7.7%)
Genotype 2		12 (6.6%)
Genotype 3		70 (38.7%)
Genotype 4		19 (10.5%)
Sofosbuvir/velpatasvir		78 (43.1%)
Glecaprevir/pibrentasvir		92 (50.8%)
Elbasvir/grazoprevir		11 (6.1%)
Prior PEG-IFN therapy		17 (9.4%)

Legend: TDF—tenofovir disoproxil fumarate; TAF—tenofovir alafenamide therapy; PEG-IFN—pegylated interferon; n/a—not applicable.

**Table 3:** Overview of the population characteristics in patients with hepatocellular carcinoma.

Variable	CHB Cohort N = 242 *n* (57.2%)	CHC Cohort N = 181 *n* (42.8%)	*p*
Hepatocellular carcinoma	12 (5.0%)	10 (5.5%)	0.697
HCC stage I	1 (0.4%)	1 (0.5%)	0.841
HCC stage II	2 (0.8%)	3 (1.6%)	0.160
HCC stage III	6 (2.5%)	2 (1.1%)	0.098
HCC stage IV	3 (1.2%)	4 (2.2%)	0.341
Vital status (deceased)	3 (1.2%)	4 (2.2%)	0.970
Time from diagnosis to death [months]	4.6 ± 3.8	7.1 ± 4.1	**0.016**
Surgical resection	7 (2.9%)	6 (3.3%)	0.947
Microwave ablation	2 (0.8%)	1 (0.5%)	0.989
TACE treatment	1 (0.4%)	0	n/a
Sorafenib therapy	5 (2.1%)	3 (1.7%)	0.997
Factors analyzed for predicting the occurrence of HCC			
Initial values of PIVKA-II [ng/mL]	129.5 (53.0–206.5)	116.0 (71.0–161.0)	0.257
Initial values of AFP [μg/L]	9.0 (2.0–6.0)	7.5 (1.0–4.0)	0.136

Legend: Statistically significant *p*-values (*p* < 0.05) are in bold.

**Table 4:** Parameters on the receiver operating characteristics curve analysis.

HCC as an Outcome Variable–CHB Cohort				
	Area under the curve	Specificity	NPV	*p*
PIVKA-II	0.809	64.5%	98.15%	**0.024**
	Cut-off	Sensitivity	PPV	Standard error
	47.0	84.5%	15.18%	0.048
	Area under the curve	Specificity	NPV	*p*
AFP	0.830	65.5%	98.31%	**0.019**
	Cut-off	Sensitivity	PPV	Standard error
	11.2	82.9%	11.91%	0.057
**HCC as an Outcome Variable–CHC Cohort**				
	Area under the curve	Specificity	NPV	*p*
PIVKA-II	0.812	61.2%	98.09%	**0.012**
	Cut-off	Sensitivity	PPV	Standard error
	46.0	82.0%	16.23%	0.006
	Area under the curve	Specificity	NPV	*p*
AFP	0.824	76.5%	97.98%	**0.020**
	Cut-off	Sensitivity	PPV	Standard error
	11.6	75.3%	15.21%	0.051

Legend: Statistically significant *p*-values (*p* < 0.05) are in bold.

**Table 5:** Results of the multivariate Cox proportional hazards model: factors associated with the development of HCC.

	**CHB Cohort**						**CHC Cohort**					
**Model 1**	**Univariate Cox Model**			**Multivariate Cox Model**			**Univariate Cox Model**			**Multivariate Cox Model**		
	**HR**	**95% CI**	***p***	**HR**	**95% CI**	***p***	**HR**	**95% CI**	***p***	**HR**	**95% CI**	***p***
Sex	1.16	0.82–1.64	0.376				1.45	0.92–2.30	0.108	1.36	0.81–2.14	0.152
Age	1.01	0.98–1.03	0.260				1.03	1.01–1.05	**0.004**	1.01	1.01–1.06	**0.012**
BMI [kg/m^2^]	0.98	0.93–1.03	0.399				1.02	0.98–1.07	0.271			
Chronic alcohol abuse	2.29	1.85–2.97	**0.036**	2.21	1.74–2.93	**0.028**	1.72	1.12–2.64	**0.013**	1.69	1.09–2.67	**0.024**
Intravenous drug use	1.35	0.78–2.34	0.281				1.58	0.93–2.67	0.086			
Non-intravenous drug use	1.12	0.72–1.75	0.614				1.21	0.74–1.99	0.438			
Smoking (current)	1.09	0.78–1.52	0.625				1.35	0.88–2.06	0.170			
F0/F1—No/mild fibrosis	1.05	0.70–1.58	0.807				1.00	0.63–1.59	0.990			
F2—Moderate fibrosis	1.22	0.78–1.90	0.372				1.43	0.85–2.41	0.177			
F3—Severe fibrosis	1.41	0.90–2.22	0.119	1.52	0.98–2.31	0.156	2.12	1.19–3.77	**0.025**	1.81	0.98–3.14	0.084
F4—Cirrhosis	1.58	0.95–2.64	0.080	1.68	0.99–2.81	0.054	3.65	2.01–6.63	**0.001**	3.60	1.98–6.35	**0.001**
FibroScan^®^ stiffness [kPa]	1.02	0.99–1.06	0.163				1.04	1.02–1.06	**0.011**	1.02	1.01–1.05	**0.008**
S0—No steatosis	1.00	0.65–1.56	0.991				1.00	0.61–1.64	0.992			
S1—Mild steatosis	0.92	0.58–1.47	0.736				0.88	0.55–1.39	0.582			
S2—Moderate steatosis	1.08	0.68–1.72	0.747				0.97	0.60–1.58	0.902			
S3—Severe steatosis	1.19	0.75–1.88	0.456				1.15	0.69–1.93	0.589			
FibroScan^®^ CAP [dB/m]	1.00	0.99–1.01	0.282				1.45	0.92–2.30	0.138			
**Model 2**	**Univariate Cox Model**			**Multivariate Cox Model**			**Univariate Cox Model**			**Multivariate Cox Model**		
	**HR**	**95% CI**	***p***	**HR**	**95% CI**	***p***	**HR**	**95% CI**	***p***	**HR**	**95% CI**	***p***
Sex	1.16	0.82–1.64	0.376	1.20	0.98–1.41	0.205	1.45	0.92–2.30	0.108			
Age	1.01	0.98–1.03	0.260	1.02	0.79–1.24	0.331	1.03	1.01–1.05	**0.004**	1.04	1.01–1.06	**0.012**
Child–Pugh class A	1.08	0.69–1.70	0.733	1.16	0.91–1.42	0.368	1.13	0.96–1.33	0.128			
Child–Pugh class B	1.25	0.80–1.95	0.325	1.23	1.00–1.45	0.527	1.21	0.90–1.63	0.084	1.14	0.87–1.98	0.159
Child–Pugh class C	1.52	0.92–2.51	0.106	1.51	1.27–1.74	0.315	1.88	1.67–2.15	**0.033**	1.64	1.21–1.81	**0.041**
Ascites	1.31	0.87–1.97	0.227	1.36	1.13–1.59	0.172	1.07	0.91–1.26	0.425			
Hepatic encephalopathy	1.28	0.81–2.01	0.281	1.29	1.03–1.55	0.466	0.99	0.83–1.19	0.966			
Portal hypertension	1.18	0.78–1.77	0.436	1.25	0.97–1.52	0.451	1.15	0.93–1.42	0.198			
Esophageal varices	1.14	0.70–1.69	0.520	1.18	0.97–1.40	0.292	1.06	0.87–1.29	0.531			

Legend: Statistically significant *p*-values (*p* < 0.05) are in bold.

**Table 6:** Results of the multivariate Cox proportional hazards model: factors associated with the development of HCC.

	CHB Cohort						CHC Cohort					
**Model 3**	**Univariate Cox Model**			**Multivariate Cox Model**			**Univariate Cox Model**			**Multivariate Cox Model**		
	**HR**	**95% CI**	***p***	**HR**	**95% CI**	***p***	**HR**	**95% CI**	***p***	**HR**	**95% CI**	***p***
Sex	1.16	0.82–1.64	0.376				1.45	0.92–2.30	0.108			
Age	1.01	0.98–1.03	0.260				1.03	1.01–1.05	**0.004**	1.04	1.01–1.06	**0.006**
Initial values of PIVKA-II	1.01	1.00–1.01	**0.004**	1.02	1.01–1.03	**0.006**	1.05	1.00–1.10	**0.015**	1.03	1.01–1.05	**0.002**
Initial values of AFP	1.13	1.11–1.17	**0.018**	1.14	1.10–1.18	**0.024**	1.11	1.03–1.20	**0.006**	1.16	1.04–1.24	**0.012**
Combined AFP/PIVKA-II	1.26	1.06–1.38	**0.001**	1.38	1.20–1.46	**0.001**	1.31	1.15–1.4	**0.001**	1.36	1.21–1.37	**0.001**
**Model 4**	**Univariate Cox Model**			**Multivariate Cox Model**			**Multivariate Cox Model**			**Multivariate Cox Model**		
	**HR**	**95% CI**	***p***	**HR**	**95% CI**	***p***	**HR**	**95% CI**	***p***	**HR**	**95% CI**	***p***
Sex	1.16	0.82–1.64	0.376				1.45	0.92–2.30	0.108			
Age	1.01	0.98–1.03	0.260				1.03	1.01–1.05	**0.004**			
HBV DNA PCR	1.98	0.65–2.54	0.620				n/a					
HBeAg positivity	1.87	1.24–2.46	**0.024**	1.56	1.17–2.31	**0.027**						
anti-HBe positivity	0.67	0.41–1.01	0.111	0.75	0.51–1.08	0.156						
TDF therapy	0.59	0.25–1.06	0.412									
TAF therapy	0.78	0.69–1.05	0.236									
HCV RNA RT PCR	n/a						1.12	0.68–1.85	0.417			
Genotype 1a							1.29	0.78–2.15	0.293			
Genotype 1b							1.41	1.01–1.96	**0.034**	1.38	1.02–2.08	**0.020**
Genotype 2							1.67	0.94–2.95	0.084	1.52	0.91–2.19	0.284
Genotype 3							1.03	0.99–1.07	0.134			
Genotype 4							0.95	0.58–1.56	0.832			
Sofosbuvir/velpatasvir							0.91	0.59–1.42	0.077			
Glecaprevir/pibrentasvir							1.02	0.66–1.57	0.938			
Elbasvir/grazoprevir							0.98	0.96–1.01	0.089	0.97	0.94–1.06	0.165
Prior PEG-IFN therapy							1.33	0.86–2.05	0.199			

Legend: Statistically significant *p*-values (*p* < 0.05) are in bold.

## Data Availability

The data supporting the reported results in this study are not publicly available due to privacy and confidentiality concerns as they consist of the patients’ medical histories. Access to these data is restricted to protect patient confidentiality and comply with ethical regulations. Requests for data access can be directed to the corresponding author, subject to institutional and ethical approvals.

## Supplementary Materials

The following supporting information can be downloaded at: https://www.mdpi.com/article/10.3390/biomedicines13112653/s1, Table S1: Laboratory parameters of the investigated cohorts; Table S2: Results of the multivariate Cox proportional hazards model: factors associated with the development of HCC.
