# Supplementary material for: Pilot Study of PIVKA-II in the Prognostic Assessment of Hepatocellular Carcinoma in Chronic Viral Hepatitis: Comparative Findings from HBV and HCV Cohorts from a Single Center in Serbia
Source: Biomedicines. 2025 Oct 29;13(11):2653. doi: 10.3390/biomedicines13112653 (PMC12649870; doi:10.3390/biomedicines13112653)
Supplement: Supplementary file 1 [file biomedicines-13-02653-s001.zip › biomedicines-3859376-supplementary.pdf]

Supplementary table 1. Laboratory parameters of the investigated cohorts

Variable	CHB cohort<br>N= 24; n (57.2%)	CHC cohort<br>N = 181; n (42.8%)	*p*
Hemoglobin [g/dL]	128 (121 – 136)	122 (115 – 130)	0.368
Platelets [×10⁹/L]	230 (190 – 280)	250 (200 – 300)	0.574
Glucose [mmol/L]	5.5 (5.0 – 6.0)	5.1 (4.6 – 5.7)	0.642
Urea [mmol/L]	6.0 (4.5 – 7.2)	5.4 (4.0 – 6.5)	0.749
Creatinine [µmol/L]	85 (75 – 100)	80 (70 – 95)	0.610
eGFR [mL/min/1.73 m²]	90 (80 – 100)	95 (85 – 105)	0.595
Sodium [mmol/L]	138 (136 – 140)	139 (137 – 141)	0.944
Potassium [mmol/L]	4.5 (4.2 – 4.8)	4.3 (4.0 – 4.6)	0.637
Total Bilirubin [µmol/L]	12 (8 – 15)	10 (7 – 14)	0.720
Direct Bilirubin [µmol/L]	3.0 (2.0 – 4.0)	2.5 (1.5 – 3.5)	0.554
AST [U/L]	25 (20 – 30)	22 (18 – 28)	0.321
ALT [U/L]	28 (22 – 35)	25 (18 – 34)	0.741
GGT [U/L]	40 (25 – 55)	30 (20 – 45)	0.682
ALP [U/L]	80 (65 – 95)	75 (60 – 90)	0.740
Total Protein [g/L]	70 (66 – 74)	72 (68 – 76)	0.920
Albumin [g/L]	43 (40 – 45)	44 (41 – 46)	0.601
INR	1.1 (1.0 – 1.2)	1.0 (0.9 – 1.1)	0.372
Prothrombin Time [sec]	13.0 (12.0 – 14.0)	12.5 (11.5 – 13.5)	0.483
Fibrinogen [g/L]	3.2 (2.8 – 3.7)	3.0 (2.6 – 3.6)	0.468
D-Dimer [mg/L]	0.4 (0.25 – 0.55)	0.35 (0.2 – 0.5)	0.142
Total Cholesterol [mmol/L]	5.0 (4.4 – 5.7)	4.8 (4.2 – 5.4)	0.851
Triglycerides [mmol/L]	1.4 (1.0 – 1.8)	1.2 (0.9 – 1.6)	0.471
Factor V [%]	100 (90 – 115)	95 (85 – 110)	0.421
Ammonia [µmol/L]	38 (28 – 48)	35 (25 – 45)	0.810

Legend: Statistically significant p-values ($p < 0.05$) are bolded.

Supplementary table 2. Results of the multivariate Cox proportional hazards model: factors associated with the development of HCC

	CHB cohort						CHC cohort					
**Model 1**	Univariate Cox model			Multivariate Cox model			Univariate Cox model			Multivariate Cox model		
	HR	95% CI	*p*	HR	95% CI	*p*	HR	95% CI	*p*	HR	95% CI	*p*
Sex	0.93	0.67 – 1.28	0.665	0.91	0.63 – 1.31	0.602	1.45	0.92 – 2.30	0.108	1.42	1.07 – 1.77	0.528
Age	1.01	0.97 – 1.06	0.640	1.02	0.97 – 1.07	0.445	1.03	1.01 – 1.05	**0.004**	1.05	1.00 – 1.22	**0.040**
Hemoglobin	1.04	0.96 – 1.14	0.345	1.01	0.93 – 1.11	0.748	0.95	0.87 – 1.03	0.205	0.88	0.67 – 1.09	0.445
Platelets	0.99	0.98 – 1.00	0.097	0.99	0.97 – 1.00	0.096	1.00	0.99 – 1.01	0.717	1.03	0.88 – 1.19	0.681
Glucose	1.00	0.98 – 1.03	0.856	1.01	0.98 – 1.04	0.423	1.01	0.99 – 1.03	0.334	1.06	0.88 – 1.25	0.176
Urea	0.98	0.92 – 1.04	0.514	0.97	0.91 – 1.05	0.368	1.03	0.97 – 1.09	0.313	0.98	0.80 – 1.15	0.396
Creatinine	1.05	0.90 – 1.23	0.522	1.03	0.87 – 1.22	0.703	1.06	0.89 – 1.26	0.522	1.05	0.86 – 1.24	0.452
eGFR	1.00	0.98 – 1.02	0.997	1.00	0.98 – 1.03	0.876	0.99	0.97 – 1.01	0.391	0.93	0.77 – 1.10	0.294
Sodium	0.98	0.94 – 1.03	0.458	0.99	0.95 – 1.04	0.642	1.01	0.97 – 1.05	0.593	1.00	0.77 – 1.23	0.188
Potassium	1.07	0.86 – 1.33	0.556	1.06	0.83 – 1.35	0.642	0.94	0.76 – 1.16	0.556	0.94	0.75 – 1.14	0.090
Total Bilirubin	0.96	0.85 – 1.08	0.477	0.95	0.83 – 1.08	0.432	1.04	0.93 – 1.17	0.460	1.06	0.88 – 1.23	0.102
Direct Bilirubin	0.94	0.77 – 1.15	0.552	0.96	0.78 – 1.19	0.703	1.07	0.89 – 1.29	0.451	1.15	0.86 – 1.43	0.577
Aspartate Aminotransferase	1.02	0.98 – 1.06	0.070	1.01	0.97 – 1.06	0.624	0.99	0.96 – 1.02	0.410	0.96	0.81 – 1.11	0.498
Alanine Aminotransferase	0.99	0.96 – 1.03	0.823	1.00	0.96 – 1.04	0.910	1.00	0.97 – 1.03	0.966	0.99	0.83 – 1.15	0.377
Gamma-GT	0.97	0.92 – 1.02	0.111	0.98	0.93 – 1.03	0.429	1.02	0.98 – 1.06	0.354	0.94	0.72 – 1.17	0.226
**Model 2**	Uniivariate Cox model			Multivariate Cox model			Univariate Cox model			Multivariate Cox model		
	HR	95% CI	p	HR	95% CI	p	HR	95% CI	*p*	HR	95% CI	*p*
Sex	0.89	0.64 – 1.24	0.495	0.92	0.65 – 1.30	0.654	1.45	0.92 – 2.30	0.108	1.36	1.05 – 1.68	0.186
Age	1.01	0.96 – 1.06	0.716	1.01	0.96 – 1.07	0.662	1.03	1.01 – 1.05	**0.004**	1.02	0.99 – 1.05	0.061
Alkaline Phosphatase	1.02	0.99 – 1.05	0.181	1.01	0.98 – 1.05	0.336	0.99	0.96 – 1.02	0.479	0.97	0.85 – 1.11	0.495
Total Protein	0.95	0.80 – 1.13	0.577	0.96	0.81 – 1.14	0.641	1.05	0.89 – 1.24	0.588	1.03	0.87 – 1.21	0.601
Albumin	1.03	0.94 – 1.13	0.529	1.02	0.93 – 1.12	0.693	0.97	0.90 – 1.06	0.486	0.96	0.83 – 1.11	0.413
INR	1.96	0.46 – 2.21	0.066	1.21	0.91 – 1.61	0.179	1.09	0.85 – 1.40	0.522	1.08	0.86 – 1.36	0.473
Prothrombin Time	1.01	0.96 – 1.06	0.824	1.00	0.95 – 1.05	0.978	0.98	0.93 – 1.03	0.437	1.00	0.88 – 1.14	0.534
Fibrinogen	0.98	0.94 – 1.02	0.301	0.99	0.95 – 1.03	0.687	1.01	0.96 – 1.06	0.756	0.99	0.85 – 1.14	0.647
D-Dimer	1.05	0.97 – 1.14	0.249	1.03	0.96 – 1.12	0.361	1.04	0.97 – 1.11	0.270	1.03	0.91 – 1.17	0.354
Total Cholesterol	1.00	0.96 – 1.04	0.920	0.99	0.95 – 1.04	0.749	0.98	0.94 – 1.02	0.298	0.97	0.86 – 1.11	0.409
Triglycerides	0.97	0.92 – 1.03	0.384	0.98	0.93 – 1.04	0.567	1.00	0.97 – 1.04	0.895	1.01	0.89 – 1.14	0.793
Ammonia	1.94	0.64 – 3.06	0.332	1.11	0.74 – 1.66	0.613	1.06	0.95 – 1.18	0.304	1.07	0.91 – 1.26	0.223

Legend: Statistically significant p-values ($p < 0.05$) are bolded.
